# National Primary Care Policy (2017) and the roles of community health workers

**DOI:** 10.1590/0034-7167-2024-0230

**Published:** 2025-01-13

**Authors:** Juliana Roza Dias, Sonia Acioli, Vanessa de Almeida Ferreira Corrêa, Inês Leoneza de Souza, Ricardo de Mattos Russo Rafael, Paulo Henrique de Almeida Rodrigues, Glaucia Bohusch

**Affiliations:** I Universidade do Estado do Rio de Janeiro. Rio de Janeiro, Rio de Janeiro, Brazil; II Universidade Federal do Estado do Rio de Janeiro. Rio de Janeiro, Rio de Janeiro, Brazil; III Universidade Federal do Rio de Janeiro. Macaé, Rio de Janeiro, Brazil; IV Universidade Federal do Estado do Rio de Janeiro, Instituto de Medicina Social. Rio de Janeiro, Rio de Janeiro, Brazil; V Universidade Federal de Santa Catarina, Colégio de Aplicação. Santa Catarina, Santa Catarina, Brazil

**Keywords:** Primary Health Care, Family Health, Public Policy, Health Policy, Community Health Workers, Atención Primaria de Salud, Salud de la Familia, Política Pública, Política de Salud, Agentes Comunitario de Salud

## Abstract

**Objective:**

To analyze the new roles of community health workers as outlined in the 2017 National Primary Care Policy (PNAB) from the perspectives of both nurses and community health workers.

**Methods:**

This qualitative study involved nurses and community health workers from Family Health teams, conducted through semi-structured interviews via videoconference between August 2021 and April 2022. The data were analyzed using thematic content analysis.

**Results:**

We identified professionals who argued that the new roles for community health workers aim to increase their autonomy and professional recognition. Others highlighted concerns about the loss of originality in their work and the potential for the illegal practice of nursing or nursing technician duties.

**Final Considerations:**

The 2017 PNAB emphasizes task execution for these workers, contributing to the mechanization of activities, reinforcing the biomedical model, and undermining the role of community health workers in promoting health within Primary Care.

## RESUMEN

**Objetivo:**

Analizar las nuevas atribuciones de los agentes comunitarios de salud preconizadas por la Política Nacional de Atención Básica (PNAB) de 2017 desde la perspectiva de los enfermeros y los agentes comunitarios de salud.

**Métodos:**

Se trata de un estudio cualitativo con enfermeros y agentes comunitarios de salud de equipos de Salud de la Familia, mediante entrevistas semiestructuradas realizadas por videoconferencia entre agosto de 2021 y abril de 2022. Los datos fueron analizados a través del análisis de contenido temático.

**Resultados:**

Identificamos profesionales que defendieron que las atribuciones de los agentes comunitarios de salud buscan aumentar la autonomía y el reconocimiento profesional de estos trabajadores. Otros señalaron la pérdida de la originalidad del trabajo y el ejercicio ilegal de la profesión de enfermero o técnico de enfermería.

**Consideraciones Finales:**

La PNAB-2017 asigna a estos trabajadores un énfasis en la ejecución de tareas, contribuyendo a la mecanización de las acciones, reforzando el modelo biomédico y desfigurando el papel del agente comunitario en la promoción de la salud en la Atención Primaria.

## INTRODUCTION

In Brazil’s Unified Health System (SUS in Portuguese), since the establishment of the Community Health Agents Program (CHAP) in the 1990s, the Family Health Program in 1994, and later with the Family Health Strategy (FHS) and previous editions of the Primary Care Policies^(1,2)^, Primary Health Care (PHC) has been selected as the central strategy for organizing and reorienting the health care model^(3)^.

The third revision of the National Primary Care Policy (PNAB in Portuguese), enacted through Ordinance GM/MS No. 2.436 on 09/21/2017^(4)^, has been widely regarded as a project to dismantle health services, receiving extensive criticism from various entities, institutions, unions, councils, and public health researchers.

Some measures that exemplify the dismantling of PHC under PNAB-2017 include the reduction in the number of Community Health Agents(CHA), flexibility in their presence within primary care teams, the flexibilization of professionals’ working hours, the removal of priority for the FHS, the termination of the Expanded Family Health and Primary Care Center (NASF-AB in Portuguese), the loss of professionals with the end of the *Mais Médicos* Program, and disincentives for a territorial approach through the new basic care funding model based on the number of registered individuals^(5,6)^, all of which undermine the organizational principles of PHC.

In addition to structural changes in PHC, PNAB introduced some controversial roles for CHA that, even when performed exceptionally, alter the nature of their work. These include measuring blood pressure, even at home, to promote health and prevent diseases; measuring capillary blood glucose, even at home, to monitor diagnosed cases of diabetes mellitus, according to the therapeutic plan prescribed by the Primary Care teams; measuring axillary temperature during home visits; performing clean dressing techniques using clean materials, running water or saline, and sterile coverings with passive dressings that only cover the wound; and providing guidance and support at home for the proper administration of medication to vulnerable patients^(4)^.

It is worth noting that the core work of CHA, since the CHAP program, has always been to serve as a link between health services and the community, based on an understanding of the social determinants of the health-disease process and the need to combine care, prevention, and health promotion activities^(7)^.

The current economic and political crises, along with significant setbacks in guaranteeing social rights, have put at risk the progress made in Brazil’s public policies, including PHC health policies, where dismantling practices are evident in this project^(8)^.

After 33 years since the creation of SUS and 30 years of the FHS, considering the current political and economic crises in the country, it is essential to reflect on the challenges and threats these policies face, as we are far from overcoming a disease-centered model focused on medical-hospital care^(9)^. However, it is important to highlight that “SUS was implemented, but it is still not consolidated”^(10)^ to the extent of significantly altering how health is delivered and how the social determinants of the health-disease process are addressed^(9)^.

Thus, the following guiding question emerged: What are the perspectives of nurses and CHA regarding the roles outlined in the 2017 - PNAB?

## OBJECTIVE

To analyze the new roles of community health workers outlined by the 2017 PNAB from the perspectives of both nurses and community health workers.

## METHODS

### Ethical aspects

This study is a segment of the thesis titled: “The 2017 National Primary Care Policy: Repercussions on the Health Practices of Nurses and Community Health Workers,” defended within the Graduate Program in Nursing at a Public University in the State of Rio de Janeiro. The study was approved by the Research Ethics Committees of a public university and a Municipal Health Department, via the Plataforma Brasil. All participants digitally signed the Informed Consent Form (ICF). To ensure participant anonymity, the letter “N” was used for nurses and “C” for CHA, followed by a numeral.

### Study type

This is a descriptive study with a qualitative approach, based on the theory of Historical and Dialectical Materialism (MHD), which is characterized by interpreting historical and social reality, emphasizing the importance of capturing a particular investigation in both the articulation and evolution of problems as well as in tracing the phenomena involved^(11)^. The Consolidated Criteria for Reporting Qualitative Research (COREQ) was used to guide the study’s methodology.

### Setting

The study was conducted in 26 Primary Health Units (PHU) within a programmatic area of a municipality in the State of Rio de Janeiro, part of the Metropolitan Health Region 1.

### Study participants

The study involved 10 nurses and 10 community health workers from Basic Health Units, composed of Family Health teams, initially selected through non-probabilistic convenience sampling. A Google Forms link was sent to the managers of the selected health units to be shared among the nurses and CHA. Due to the low response rate, snowball sampling was used, with contact being established via participants’ cell phones.

As inclusion criteria, participants were selected if they were part of the functional staff of a specific programmatic area and were active during the data collection period. Those on vacation, medical leave, or otherwise absent from their professional activities during the data collection period were excluded from the study.

The number of participants was determined by response saturation, identified through the redundancy and repetition of information^(12)^.

### Data Collection and Organization

Data collection took place between August 2020 and April 2021 through semi-structured interviews conducted via videoconference. The interviews were carried out by the lead author of this manuscript, a nurse who, at the time, was a doctoral student in a Graduate Program at a public university in the State of Rio de Janeiro. The interviews were scheduled outside the healthcare professionals’ working hours to ensure privacy and confidentiality. During the interviews, participants were given the option to keep their cameras turned off, allowing only the audio to be recorded. The interview script was structured around three thematic axes: the repercussions of PNAB-2017 on health practices; PNAB-2017 and health care models; and PNAB-2017 and municipal management. The interviews lasted an average of 40 minutes. It is worth noting that the script was subjected to a pilot test with three healthcare professionals working in Family Health teams, with no need for modifications to its structure.

### Data Analysis

The participants’ responses were analyzed using Bardin’s content analysis technique^(13)^, following the stages of pre-analysis, material exploration, and interpretive synthesis of the results. The data were processed by transforming raw information into meaningful results. The findings were grouped into categories based on thematic similarity, with the percentage of occurrence calculated from 947 Units of Registration (UR). Three thematic categories were identified: (1) Structural and functional changes in PHC; (2) Implications of the restructuring of the work process, roles, and organization of community health workers in PHC; and (3) Repercussions of PNAB-2017 for users and the FHS model.

In this article, we present Category 2, which addresses the proposed objective and emerged from a total of 309 UR, corresponding to 32.63% of the analysis, presented through 80 Units of Significance. The data were organized using Microsoft Excel.

## RESULTS

From the analysis of the interviews, different perspectives were identified among the study participants: on one side, those in favor, and on the other, those opposed to the changes in the roles of CHA as outlined in PNAB-2017.

The professionals in favor, both nurses and CHA, justified their stance by highlighting the potential for faster diagnosis and early treatment of diseases, which could contribute more effectively to the team’s workflow. They also considered factors such as the reduced number of nursing technicians and nurses compared to CHA in the units, the distance between health units and micro-areas, the difficulty in accessing the homes of bedridden patients, and the importance of training community health workers to perform these procedures.

*I think it’s good, I think,* [...] *I’m not against it at all,* [...] *in my unit, there are twenty-five CHA and eight technicians* [...] *sometimes one of them gets sick, sometimes they’re busy administering medication, and we can check blood pressure, measure glucose, as long as we’re trained for that* [...]. (C1)

*With the bedridden patient, and their blood pressure was high, the technician couldn’t leave. I had to stop everything I was doing, go up there-it’s far-to check vital signs. To make a diagnosis, you need at least two readings, and I had to go up there. I said: ‘I’ll just go myself', rushed, checked, ‘blood pressure is still high', and then fifteen days passed before I could go back up to check again. If the CHA had that skill, they could do it. I know they’re capable and were well-trained. They could do it, and I’d get faster feedback so the doctor could start the medication* [...]. (N3)

[...] *It helps with the identification process of a problem. When they come to us, they already bring something more detailed about what we can do for treatment.* [...] *I’m in favor; it has a great impact because, in the previous PNAB, the health agent didn’t have this responsibility.* (N10)

It is observed that these technical procedures are still not authorized by municipal management. The CHA report fear of retaliation and/or suspension from their superiors when performing the new tasks, despite being in favor of their implementation, provided they receive proper training for these functions. However, some nurses mention that only CHA with nursing technician training sporadically and informally measure the blood pressure of some users.

[...] *They would do it with complete calm and care, without any problem; they just don’t do it because local management hasn’t approved it, you understand?* [...] *If they said: ‘Look, you can take blood pressure, you can take the equipment',* [...] *they would do it without any problem. But now, they are afraid of doing these things and being reprimanded or warned* [...]. [...] *I’ve even told them: ‘But it’s me asking you, it’s not the manager, it’s not the unit', but they claim they are not authorized to do it* [...]. (N8)

[...] *A community health worker who has more empathy for the patient will go ahead and check vital signs because they are also a nursing technician, even though they don’t get paid extra for being a technician. Since they already have the knowledge and empathy for the patient, they go ahead and do the extra work during home visits. But if they don’t have that empathy and the patient is just another regular patient, they will bring the information to the team nurse or to a nursing technician for a follow-up visit.* (N10)

The CHA participants highlight that performing these procedures is seen as an opportunity to increase their autonomy and professional recognition. However, they also express discomfort with the excessive bureaucratic tasks assigned to them, which do not lead to proper recognition of their profession. On the other hand, both groups of professionals acknowledge that some of these responsibilities belong to nursing technicians and emphasize the need for proper qualifications and/or training to carry out these procedures.

[...] *In terms of potential, I think it’s the fact that they feel more secure and useful, the independence of not having to wait for the nurse* [...] *to perform this task. The right word is autonomy. To make a visit with a bit more quality. When you arrive at the patient’s home and bring the equipment to measure their blood pressure and glucose, they feel more valued, as if they were in a consultation* [...]. (N6)

*I think it’s extremely important. If they offered courses* [...] *not just telling them: ‘You’re going to take blood pressure, do a dressing, and that’s it', no, they should train the health workers properly so that we can perform these tasks. It’s very important. I would be honored to arrive and take the user’s blood pressure. Often, the patient can’t come here to see the nurse or the doctor, and it would reduce the workload for the nurse and doctor if the health worker could do the visit*. (N10)

Another important point highlighted by the participants in favor of the changes is the recognition of the technical nursing training required for the legal execution of such duties. They consider that, since many CHA have technical training or are nursing students, they could perform these procedures, thus legitimizing these practices in their daily routine. However, they overlook another legal issue, which is the risk of role deviation, as the professional would still be hired as a community health worker.

*Most of my CHA, except for one, are nursing technicians. They have a technical degree, and many of them are in nursing school, but even so, they are not allowed to perform these tasks, which I think is wrong. If they are visiting the patient, it wouldn’t hurt for them to carry a blood pressure monitor or a glucose meter, because if, say, the patient’s glucose levels are significantly altered, we can go there, administer insulin, give medication, or check the patient’s chart to see what’s needed, you know? It would really help. This idea is very good, it would speed up the work a lot, really a lot*. (N8)

[...] *Many of us already have the knowledge, many of us are already in the health field, and many are nursing technicians. However, even they are not authorized to perform these types of services* [...]. (C4)

Nurses who are against the inclusion of the tasks outlined by the PNAB for CHA mentioned various factors, including legal issues related to professional practice, role deviation, the need for technical training, the fragility of working conditions and workloads, concerns about the lack of support in the supervision of nurses, noting that CHA are not part of the nursing team, and the potential loss of the CHA’s role in primary care.

[...] *Even my CHA are nursing technicians. Where I work, they receive a bonus from the company for that, but I tell them: “Here you are hired as CHA* [...] *don’t do it because it’s not part of your role. We have a nursing technician, so she will do the dressing, she will administer the medication. If I find out you’re doing it, I will issue a warning".* (N11)

[...] *I understand the labor issues, but they would come through ordinances, they would be regulated, and they would receive training. So, there are bureaucracies for this to happen, you know? And it could happen. The question is, how will it impact the work at the unit, their personal and professional lives, and our control, given the situations we face daily at the unit.* (N4)

[...] *Coren has not made any statement about this yet, nor has COFEN, so before implementing this policy, there needs to be a discussion among the professional entities, in this case, nursing. Who is responsible for the dressing? The nurse. So, if the CHA does it and uses a dressing that worsens the patient’s wound, who is accountable? The nurse supervising the CHA’s work? Ok, but are they supervising the dressing in that case? I think there is a gap, and while that gap exists, I think it’s better for us not to do it, because we don’t have the necessary support* [...]. (N11)

Regarding labor issues, the study participants expressed concerns about the illegal practice of the nursing technician profession and the role deviation of CHA, even for those with nursing technician training, as they were not hired for this function.

Professionals who oppose the execution of procedures and techniques by CHA argue that nursing technician training is necessary to ensure the safe and legal performance of these duties. Thus, mere training or qualifications would not be sufficient, given the technical and scientific complexity involved. It is essential to preserve the quality of care and the safety of the patient. Furthermore, even if a CHA has technical training and qualifications to perform these procedures, it would still be considered a role deviation. Another risk mentioned is the supervision of these actions, as there is insufficient support for the team nurse, given that CHA are not part of the nursing professional category.

*I am against it because they don’t have technical training and haven’t completed a nursing technician course, and the most a CHA can have is a technical CHA course.* [...] *You need to have all the technical and scientific knowledge for that technique,* [...] *to be a CHA, only a high school level is required, so I think putting that responsibility on a professional with only a high school education is a lot.* (N11)

[...] *Incorrectly measuring blood pressure can also harm someone’s life. It’s not just about putting the cuff on and that’s it; there’s a whole technique involved. So, I’m against it, and if I can,* [...] *this won’t happen on my team because we have a qualified professional, the nursing technician, who is there to perform non-invasive procedures, dress wounds, check glucose levels, and measure blood pressure. That’s what they’re there for, and I’m completely against it.* (N11)

From this perspective, nurses who oppose incorporating the new roles outlined by PNAB-2017 for CHA also mentioned the worsening of working conditions and workloads, already exacerbated by the reduction in the number of CHA on teams, leading to an accumulation of duties, inadequate salaries related to the new roles, and a negative impact on the routine and work processes of the Family Health team.

[...] *It’s a professional who doesn’t have a decent salary, a salary at a high school level, and you’re going to give them more responsibilities on top of what they already have. If we think about it, there’s no obligation for a set number of them, so let’s say one CHA is doing the work of two or three people and still has to do all this-it’s exhausting.* (E1)

[...] *No, no, and no. If I live in a bubble, I want to stay in that bubble, but we don’t do that there, no way.* [...] *I think it’s an unnecessary accumulation of duties, and supervising it would also be very complicated. Just imagine, someone seeing a CHA performing a procedure, then that person lives in the community, and it’s a Saturday or Sunday, and they go to the CHA’s house asking them to do that procedure. I’m sorry, but people don’t have common sense. I think it would be terrible for their personal life too. It’s already bad, and I think it would get exponentially worse.* (E4)

Participant N11 argues that PNAB-2017 positions CHA as task executors, contributing to the mechanization of their actions, reinforcing the biomedical model, and distorting their role in PHC.

[...] *So, it’s not just about arriving and mechanizing everything. What I think is that the person who formulated this thinks that way-that it’s just about measuring blood pressure. Even to measure blood pressure, you have to know how to do it. You have to know how to guide the patient: ‘Did you walk here? You need to wait. Did you smoke? Did you drink water?* [...]*'. So, I think it was a very mechanical way of viewing the CHA as just an executor, performing tasks without critical thinking* [...]. (N11)

Additionally, we found reports from study participants referring to the resistance of CHA to incorporating the new tasks into their practices within the Family Health team. Consequently, it was identified that some study participants who reject PNAB-2017 support the previous policy. They emphasize that measuring blood pressure, glucose, and temperature, as well as dressing wounds, are nursing tasks and do not belong to the role of CHA, once again pointing to the increased workload in monitoring users.

[...] *But they are completely against it. 99% are against it. It’s been discussed in meetings, and everyone was against it. It was a terrible mess. No one accepts this idea of going to the patient’s house and taking their blood pressure.* (C3)

*That’s a task for the nursing technician, not the community health worker. It’s their role because the technician has a day in the week dedicated to visits in the community, in the territory* [...]. *I don’t think this is a CHA task* [...]. (C6)

The nurses interviewed emphasize that CHA are essential in the territory for building relationships, conducting active searches, welcoming patients, and detecting users’ health needs, which is their true role. These new tasks distance CHA from their original function, which could create gaps in care and have negative repercussions on the teams’ workflow.

[...] *I need their perception, not for them to dress wounds. I think we miss the point when it comes to their tasks because that’s not what we lack. What we really need is for them to provide excellent care, which is what they were born to do-to be able to identify each patient individually. Just because one patient is like this doesn’t mean another with the same story will be treated the same. We need to understand that each human being is unique*. [...] *They need to make themselves indispensable because, to me, they are. If I didn’t have CHA on my team, I wouldn’t know how to work because they’re the ones who filter the demands that come in. They filter the lies, and they can identify urgent situations.* (N9)

## DISCUSSION

In September 2017, the Federal Nursing Council (COFEn in Portuguese) requested clarification from the Ministry of Health (MH) regarding the performance of nursing technical procedures by community health workers, as outlined by PNAB-2017^(14)^. During the same period, a COFEn plenary session was held with representatives from the MS Department of Primary Care and the National Council of Health Secretaries (CONASS in Portuguese) to discuss the new responsibilities assigned to community health workers, which are covered under Law 7.498/1986^(15)^, regulating the nursing profession. However, then Minister of Health, Ricardo Barros, stated that only agents with nursing technician training would perform such tasks. This statement drew criticism from the council members, as there was no assurance of requiring nursing technician qualifications^(14)^.

In 2018, COFEn met with the National Confederation of Community Health Workers and Endemic Disease Control Agents (CONACS in Portuguese) to discuss the role deviation imposed by the MS through PNAB-2017, asserting that this policy mischaracterized the roles of CHA and aimed to use them as nursing technicians or even phase out the profession, weakening their role in SUS and PHC, potentially posing risks to public health^(16)^.

It is important to highlight two other MH ordinances that, due to pressure and resistance from professionals, were later revoked: Ordinance No. 958/2016^(17)^, which proposed replacing CHA with nursing assistants or technicians, and Ordinance No. 83/2018^(18)^, which proposed technical nursing training for community health workers and endemic disease control agents through the Nursing Technician Training Program for Health Agents (PROFAGS in Portuguese).

According to the Brazilian Nursing Association (ABEn in Portuguese), the PROFAGS program distorts the professional roles in PHC by making the work processes in Primary Care more flexible and mischaracterizing them, in addition to threatening the jobs of community health workers^(19)^. COFEn’s Health Agents Working Group issued a statement opposing PROFAGS, arguing that if CHA were trained as nursing technicians, they should be hired for that specific role and not as community health workers^(20,21)^.

Although these ordinances were revoked due to resistance from professionals and scientific entities such as ABEn, CONACS, and COFEn, the tasks were consolidated in Ordinance GM/MS No. 2.436/2017^(4)^. After analyzing the roles and legislation related to CHA and nursing technicians, it was determined that the ordinances reflected the idea that CHA would no longer be necessary in PHC, as they were considered insufficiently effective in addressing chronic-degenerative diseases. It was suggested that nursing technicians would better meet these demands, rendering CHA dispensable in the minimum composition of teams^(22)^. This conclusion is reinforced by PNAB-2017, which makes the presence of CHA optional in Primary Care teams^(4)^.

We agree that both ordinances reinforce the ideas of PNAB-2017, as they were implemented, highlighting a bold plan to dismantle SUS. These ordinances consistently focus on the tension between the public and private sectors, treating health as a commodity and approaching Primary Care in a selective and reduced manner, aligned with the logic of the biomedical and hegemonic model^(23)^.

The execution of nursing-specific tasks by CHA will create administrative, legal, and management problems for PHC, such as a shortage of CHA or nursing technicians in the units-professionals essential for the functioning of fundamental activities. These are some of the barriers that lead to role deviation and the confinement of CHA to health units^(19)^.

According to the Nursing Practice Law (Law 7.498/1986)^(14)^, nurses supervise and are ethically responsible for the actions of nursing assistants, nursing technicians, and midwives, but CHA are not part of the nursing team. However, regarding the responsibilities of CHA and endemic disease control agents (DCA), PNAB-2017 defines both common and specific duties for these categories, including supervision by higher-level professionals, such as the team nurse^(4)^.

Technical opinion COREn-DF No. 051/CTA/2022, in response to questions about who should supervise the activities of public health agents in PHC and regarding the teaching and execution of nursing techniques and procedures, reaffirms that supervision must be carried out in conjunction with the multiprofessional team. However, the opinion ambiguously concludes by allowing CHA to perform techniques and procedures in accordance with the guidelines of PNAB-2017, which contradicts the positions of COFEn and ABEn^(20)^.

In light of the various attacks on PNAB, especially regarding the optional presence of CHA in Primary Care teams, the reduction of their numbers in FHS, and the inclusion of new responsibilities for these workers-who are already burdened with educational and health promotion practices-there is a risk of setbacks in PHC workflows. This could obscure the life realities that influence health-disease processes^(24,25)^.

This perspective is also mentioned in another study that advocates for the inclusion of nursing technicians and assistants in Family Health teams. It is further emphasized that incorporating nursing practices into the work of CHA results in an increase in technical procedures in the field, to the detriment of the essential activities carried out by CHA^(7)^.

As a member of the interprofessional team, the CHA contributes in various ways to the promotion, prevention, investigation, diagnosis, and recovery of the population’s health. This role highlights the need for a cohesive health team with intersectoral initiatives to address the diverse demands of the territory in which they operate^(24)^.

Over the years, the practices of CHA have evolved, bringing new challenges, including facing resistance and fighting for better working conditions, as well as maintaining their original role focused on health promotion and prevention within the territory. According to the CHA themselves, these actions were seen as responsible for gaining recognition and respect from users. However, their health practices have become increasingly distant from users due to excessive administrative tasks and prolonged stays inside the health units. This distance from the territory prevents proper follow-up with families, which undermines the work of CHA and compromises the objectives of the FHS^(26)^.

It is evident that privatization and mercantilist interests, which became dominant in the federal government between 2015 and 2022, influenced the formulation of a series of projects, constitutional amendments, and public statements aimed at weakening SUS and replacing it with low-quality private services. These services are based on “other” models of care, with limited “service packages,” similar to health insurance, which follows the biomedical model^(19)^.

The transfer of nursing technician responsibilities to CHA also points to cost reduction, as maintaining these workers is less expensive than increasing the number of technicians. This favors the capitalist logic in the health sector, leading to the deterioration of services and working conditions.

Under the hegemony of neoliberal rationality, privatization has increasingly infiltrated SUS, gradually becoming part of the system’s framework. Market-oriented ideas have been incorporated into policy designs and management processes, imposing limits on the universalization of rights, the expanded conception of health, and the financial foundation of social security^(25)^.

The relationship between power and knowledge, linked to the social practice of CHA, has changed with the institutionalization of these workers, distancing them from their original functions, roles, and profiles, especially due to the technical influences in health, where knowledge is altered and power becomes a contested object. Thus, the public integration, the creation of the CHA profession, and the regulation of their work have redefined their functions, roles, and responsibilities^(27)^.

The formation of CHA is centered on popular knowledge, with the aim of promoting health through popular participation, which enhances the understanding that health is a social right shaped by the social determinants of health. As such, their training does not have a historical foundation based on the traditional biomedical model^(28)^.

In this context, the precarization of healthcare intensifies with the replacement of these professionals by nursing assistants or technicians, as outlined in Ordinance 958/2016^(17)^, and with the emergence of PNAB-2017, which makes the presence of CHA optional in Primary Care teams, reducing their participation in Family Health teams and incorporating nursing procedures into the CHA role.

The repercussions of PNAB-2017, along with the contradictions and concerns identified in this study, reveal the concrete reality of CHA health practices. It is understood that the adoption of the neoliberal model in health services is linked to the precarization of healthcare workers, including CHA and nurses, with the implementation of low wages, poor working conditions, the degradation of labor relations, and a reduction in care teams^(29)^. This contributes to the erosion of these professionals’ health practices.

This is a political movement that undermines healthcare by devaluing its workers, putting at risk the proposal of continuous therapeutic relationships and the maintenance of the principles of territoriality and community care, which are pillars of PHC and are being lost with the reorganization of the system^(29)^.

These changes reflect the contradictions in the relationship between CHA and their work, polarizing and confusing the professionals themselves regarding their origins, roles, and responsibilities, while also threatening their existence and importance in health services. This depletion of the profession can be associated with the realization of neotaylorism, which demands higher education and qualifications for certain professions, such as the nursing technical duties assigned to CHA. However, it is evident that while some professions are gaining new responsibilities, others are being depleted^(30)^.

### Study Limitations

One limitation of the study is the exclusion of other Programmatic Areas in the city of Rio de Janeiro, as well as the small number of research participants in the selected AP, exacerbated by the health crisis caused by the COVID-19 pandemic. Another limitation was the exclusion of other professionals, managers, and FHS users from this study. However, despite these limitations, this study provides an overview of professionals’ perceptions regarding the new responsibilities of CHA. It is considered that there are risks to public health when professionals are allowed to perform technical procedures without the proper training and competence, weakening both PHC and FHS.

### Contributions to the Field

This study highlights the need for further reflection and in-depth research, utilizing other methods, on the roles and contributions of CHA in Family Health teams, reinforcing and restoring the role of this professional in this field of care. It is crucial that nursing professionals play an active role in enforcing the Nursing Practice Law and promote discussions, reflections, and mobilizations to restructure PNAB according to SUS principles. The goal should be to strengthen and expand PHC, prioritizing FHS, while primarily respecting the scope of responsibilities, competencies, and skills based on the training of health team professionals.

## FINAL CONSIDERATIONS

Given the polarization between nurses and CHA who are either in favor of or opposed to the inclusion of nursing technician tasks for CHA, it is essential to broaden the debate on the social and economic recognition of this work, which is the reasoning behind why some professionals agree with the new responsibilities.

It is necessary to consider the different contexts and contradictions present in CHA health practices. The need to fulfill predetermined tasks ends up shaping new forms of service delivery, which do not always take into account the loss of social rights, such as the right to health. It seems there has not been enough reflection on the quality of care, patient safety, access, and comprehensive care. Comments such as “not getting overwhelmed” and “properly dividing users among the CHA” are responses to a policy that leads health professionals to accept what is imposed, revealing our limited participation in health decision-making processes and our “lack of strength” in defending health as a right for all.

The current policy positions CHA as mere task executors, contributing to the mechanization of actions, the precarious working conditions for CHA, reinforcing the biomedical model, and distorting their role in health promotion within PHC.
