# Supplementary material for: National Primary Care Policy (2017) and the roles of community health workers
Source: Rev Bras Enferm. 2025 Jan 13;78(1):e20240230. doi: 10.1590/0034-7167-2024-0230 (PMC11731852; doi:10.1590/0034-7167-2024-0230)
Supplement: Translation

# Translation: Política Nacional de Atenção Básica (2017) e as atribuições dos agentes comunitários de saúde

**DOI:** 10.1590/0034-7167-2024-0230pt

**Authors:** Juliana Roza Dias, Sonia Acioli, Vanessa de Almeida Ferreira Corrêa, Inês Leoneza de Souza, Ricardo de Mattos Russo Rafael, Paulo Henrique de Almeida Rodrigues, Glaucia Bohusch

**Affiliations:** I Universidade do Estado do Rio de Janeiro. Rio de Janeiro, Rio de Janeiro, Brasil; II Universidade Federal do Estado do Rio de Janeiro. Rio de Janeiro, Rio de Janeiro, Brasil; III Universidade Federal do Rio de Janeiro. Macaé, Rio de Janeiro, Brasil; IV Universidade Federal do Estado do Rio de Janeiro, Instituto de Medicina Social. Rio de Janeiro, Rio de Janeiro, Brasil; V Universidade Federal de Santa Catarina, Colégio de Aplicação. Santa Catarina, Santa Catarina, Brasil

**Keywords:** Atenção Primária à, Saúde, Saúde da Família, Política Pública, Política de Saúde, Agentes Comunitários de Saúde

## Abstract

**Objetivo:**

Analisar as novas atribuições dos agentes comunitários de saúde preconizadas pela Política Nacional de Atenção Básica (PNAB) de 2017 sob a ótica dos enfermeiros e dos agentes comunitários de saúde.

**Métodos:**

Estudo qualitativo com enfermeiros e agentes comunitários de saúde de equipes de Saúde da Família, por meio de entrevistas semiestruturadas entre agosto de 2021 e abril de 2022. Os dados foram submetidos à análise de conteúdo temática.

**Resultados:**

Identificamos profissionais que defenderam que as atribuições dos agentes comunitários visam o aumento da autonomia e valorização profissional desses trabalhadores. Outros apontaram para a perda da originalidade do trabalho, para o exercício ilegal da profissão de enfermeiro ou de técnico de enfermagem.

**Considerações Finais:**

A PNAB-2017 atribui a esses trabalhadores uma ênfase na execução de tarefas, contribuindo para a mecanização das ações, reforçando o modelo biomédico e descaracterizando o papel do agente comunitário na promoção da saúde na Atenção Primária.

## INTRODUÇÃO

No Sistema Único de Saúde (SUS) brasileiro, desde o estabelecimento do Programa de Agentes Comunitários de Saúde (PACS) nos anos 1990, do Programa de Saúde da Família (PSF) em 1994, e posteriormente, com a Estratégia Saúde da Família (ESF) e nas edições anteriores das Políticas de Atenção Básica^(1,2)^, a Atenção Primária à Saúde (APS) foi eleita como a estratégia central na organização e reorientação do modelo de atenção à saúde^(3)^.

A terceira revisão da Política Nacional de Atenção Básica (PNAB), promulgada por meio da Portaria GM/MS nº 2.436 de 21/09/2017^(4)^, considerada por muitos como um projeto de desmonte da saúde, foi aprovada diante de várias críticas por parte de diversas entidades, instituições, sindicatos, conselhos e pesquisadores do campo da Saúde Coletiva.

Podemos citar algumas medidas que exemplificam o desmonte da APS promovido pela PNAB-2017, como a redução do número de agentes comunitários de saúde, flexibilizando sua presença nas equipes de atenção básica; a flexibilização da carga horária dos profissionais; a abolição da prioridade para a ESF; a extinção do Núcleo Ampliado de Saúde da Família e Atenção Básica (NASF-AB); a perda de profissionais com o encerramento do Programa Mais Médicos; e os desincentivos à abordagem territorial com o novo modelo de financiamento da atenção básica, baseado no número de cadastrados^(5,6)^, o que descaracteriza os princípios organizativos da APS.

Além das alterações estruturais na APS, a PNAB acrescenta algumas polêmicas atribuições aos agentes comunitários de saúde que, mesmo quando executadas de forma excepcional, descaracterizam as práticas de saúde desses trabalhadores. Entre elas estão: aferir a pressão arterial, inclusive em domicílio, com o objetivo de promover saúde e prevenir doenças e agravos; realizar a medição da glicemia capilar, inclusive em domicílio, para acompanhamento de casos diagnosticados de diabetes mellitus, conforme o projeto terapêutico prescrito pelas equipes da Atenção Básica; aferição da temperatura axilar durante a visita domiciliar; realizar curativos com técnicas limpas, utilizando material limpo, água corrente ou soro fisiológico e cobertura estéril, com uso de coberturas passivas, que apenas cobrem a ferida; e oferecer orientação e apoio, em domicílio, para a correta administração da medicação ao paciente em situação de vulnerabilidade^(4)^.

Vale ressaltar que a essência do trabalho do agente comunitário de saúde (ACS), desde o PACS, sempre foi a articulação entre os serviços de saúde e a vida no território, a partir da compreensão dos determinantes sociais do processo saúde-doença e da necessidade de conjugar ações de cuidado, prevenção e promoção da saúde^(7)^.

O contexto de crise econômica e política, e os eminentes retrocessos no que diz respeito à garantia de direitos sociais, têm colocado em risco os avanços obtidos nas políticas públicas no Brasil, inclusive na política de saúde da APS, onde as práticas orientadas para o desmonte são visíveis neste projeto de desmantelamento^(8)^.

Passados 33 anos da criação do SUS e 30 anos da ESF, diante do contexto de crise política e econômica do país, é necessário refletir sobre os desafios e ameaças que essas políticas enfrentam, uma vez que estamos longe de superar o modelo centrado na doença e na assistência médico-hospitalar^(9)^. No entanto, vale ressaltar que “o SUS foi implantado, mas ainda não está consolidado”^(10)^ a ponto de alterar de forma expressiva o modo de produzir saúde e de enfrentar os determinantes sociais do processo saúde-doença^(9)^.

Dessa forma, delineou-se a seguinte questão norteadora: Qual é a visão dos enfermeiros e agentes comunitários de saúde acerca das atribuições preconizadas pela PNAB de 2017?

## OBJETIVO

Analisar as novas atribuições dos agentes comunitários de saúde preconizadas pela PNAB de 2017 sob a ótica dos enfermeiros e dos agentes comunitários de saúde.

## MÉTODOS

### Aspectos éticos

Este estudo é um recorte da tese intitulada: “A Política Nacional de Atenção Básica 2017: repercussões nas práticas de saúde de Enfermeiros e Agentes Comunitários de Saúde”, defendida no Programa de Pós-Graduação em Enfermagem de uma Universidade Pública do Estado do Rio de Janeiro. O estudo foi aprovado pelos Comitês de Ética em Pesquisa (CEP) de uma universidade pública e de uma Secretaria Municipal de Saúde, por meio da Plataforma Brasil. Os participantes assinaram digitalmente o Termo de Consentimento Livre e Esclarecido (TCLE). Para assegurar a anonimização dos participantes, utilizou-se a letra E para enfermeiros e A para ACS, seguida de um numeral.

### Tipo de estudo

Trata-se de um estudo descritivo, com abordagem qualitativa, fundamentada na teoria do Materialismo Histórico e Dialético (MHD), que se caracteriza pela interpretação da realidade histórica e social, enfatizando a importância da captação de uma determinada investigação, tanto nas articulações e evoluções dos problemas quanto no rastreamento dos fenômenos envolvidos^(11)^. O instrumento *Consolidated Criteria for Reporting Qualitative Research* (COREQ) foi adotado para nortear a metodologia do estudo.

### Cenário

O estudo foi desenvolvido em 26 Unidades de Atenção Primária (UAP) de uma área programática de um município do Estado do Rio de Janeiro, pertencente à Região de Saúde Metropolitana 1.

### Participantes do estudo

Participaram do estudo 10 enfermeiros e 10 agentes comunitários de saúde de Unidades Básicas de Saúde constituídas por equipes de Saúde da Família, selecionados inicialmente por meio de amostragem não probabilística por conveniência, através do envio de um *link* para um formulário do *Google Forms* aos gerentes das unidades de saúde selecionadas, para que fosse compartilhado entre os enfermeiros e ACS. Diante do baixo número de respostas, recorreu-se à amostragem em formato de “bola de neve”, estabelecendo contato por meio do telefone celular de cada participante.

Como critério de inclusão, foram selecionados para o estudo os participantes que faziam parte do quadro funcional de uma determinada área programática e que estiveram atuantes durante o período de coleta de dados. Foram excluídos aqueles que, durante o período de coleta de dados, estavam de férias, em licença-saúde ou afastados de suas atividades profissionais por qualquer motivo.

O número de participantes foi definido por saturação das respostas, identificada a partir da redundância e repetição de informações^(12)^.

### Coleta e organização dos dados

A coleta dos dados foi realizada entre agosto de 2020 e abril de 2021, por meio de entrevistas semiestruturadas conduzidas por videoconferência. As entrevistas foram realizadas pela autora principal deste manuscrito, enfermeira e, à época, doutoranda em um Programa de Pós-Graduação de uma universidade pública do Estado do Rio de Janeiro. As entrevistas ocorreram fora do horário de trabalho dos profissionais de saúde, visando garantir a privacidade e o sigilo das informações. Durante as entrevistas, foi oferecida ao participante a opção de manter a câmera desligada, permitindo a gravação apenas do áudio. O roteiro da entrevista foi estruturado em três eixos temáticos, abordando: as repercussões da PNAB-2017 nas práticas de saúde; a PNAB-2017 e os modelos de atenção à saúde; e a PNAB-2017 e a gestão municipal. As entrevistas tiveram uma média de duração de 40 minutos. Vale ressaltar que o roteiro foi submetido a um teste piloto realizado com três profissionais de saúde que atuavam em equipes de Saúde da Família, sem necessidade de modificações em sua estrutura.

### Análise dos dados

As falas dos participantes foram submetidas à técnica de análise de conteúdo de Bardin^(13)^, seguindo as etapas de pré-análise, exploração do material e síntese interpretativa dos resultados. O tratamento dos dados consistiu na transformação das informações brutas em resultados significativos. Os achados foram agrupados em categorias por similaridade temática, com cálculo de percentual de ocorrência, gerado a partir de 947 Unidades de Registro (UR). Três categorias temáticas foram identificadas: (1) Alterações estruturais e funcionais da APS; (2) Implicações da reestruturação do processo de trabalho, atribuições e organização do agente comunitário de saúde na APS; e (3) Repercussões da PNAB - 2017 para os usuários e para o modelo da ESF.

Neste artigo, apresentamos a categoria 2, que responde ao objetivo proposto e emergiu de um total de 309 UR, correspondendo a 32,63% da análise, apresentadas por meio de 80 Unidades de Significação. Os dados foram organizados no *Microsoft Excel*.

## RESULTADOS

A partir da análise das entrevistas, identificaram-se diferentes visões entre os participantes do estudo: de um lado, aqueles favoráveis, e de outro, os contrários às mudanças nas atribuições dos ACS preconizadas pela PNAB-2017.

Os profissionais favoráveis, tanto enfermeiros quanto ACS, basearam suas justificativas na possibilidade de maior celeridade no diagnóstico e no tratamento precoce de doenças, como uma forma de contribuir efetivamente para o processo de trabalho da equipe. Consideraram, ainda, as seguintes situações: o número reduzido de técnicos de enfermagem e enfermeiros em comparação com os ACS nas unidades; a distância entre as unidades de saúde e as microáreas; a dificuldade de acesso às residências dos usuários acamados; e a importância do treinamento dos agentes comunitários para a realização dos procedimentos.

*Eu acho legal, eu acho,* [...] *eu não sou contra não,* [...] *na minha unidade tem vinte e cinco ACS e oito técnicos* [...] *às vezes um fica doente, às vezes está cheio para aplicar uma medicação, e a gente pode aferir uma pressão, verificar uma glicemia, com tanto que a gente esteja treinado para isso* [...]. (A1)

*No acamado e ele estava com a pressão alta, o técnico não conseguia sair, eu tinha que me deslocar cheia de coisa para fazer, ir lá em cima, é longe, para poder verificar sinais vitais, para dar o diagnóstico tinha que ter pelo menos duas aferições e eu tive que subir, falei: “vou eu mesmo” corre, verifica, “tá de alta de novo”, se passaram quinze dias para eu conseguir subir para verificar, se o ACS tem essa habilidade ele faria isso, eu sei que ele está apto, que foi bem treinado, ele conseguiria fazer isso e eu conseguiria um retorno mais rápido para que o médico conseguisse iniciar o medicamento* [...]. (E3)

[...] *facilita no processo de identificação de algum problema. Quando já vem para a gente já vem alguma situação um pouco mais detalhada do que a gente pode fazer de conduta.* [...] *Eu sou a favor, isso tem um grande impacto porque na PNAB anterior a essa, o agente de saúde não tinha essa questão*. (E10)

Observa-se, a seguir, que a realização desses procedimentos técnicos ainda não está autorizada pela gestão municipal. Os ACS relatam medo de retaliações e/ou suspensões por parte de seus superiores ao exercerem as novas atribuições, embora sejam favoráveis à sua implementação, desde que recebam o treinamento adequado para essas funções. No entanto, alguns enfermeiros mencionam que apenas os ACS que possuem formação técnica em enfermagem realizam, de maneira esporádica e informal, a aferição da pressão arterial de alguns usuários.

[...] *por eles, eles fariam com a maior tranquilidade e carinho sem problema algum, eles só não fazem porque a gerência do local não libera, entendeu?* [...] *Se falassem: ó, vocês podem aferir, vocês podem levar o aparelhinho,* [...] *eles fariam com a maior tranquilidade, sem problema nenhum, agora, eles têm medo de fazerem as coisas e serem chamados e advertidos* [...]. [...] *eu já até falei: “mas sou eu que estou te pedindo, não é a gerente, não é a unidade”, mas eles alegam que isso eles não são liberados a fazer* [...]. (E8)

[...] *agente de saúde quando tem uma empatia a mais pelo paciente faz o que é estabelecido de fazer a verificação dos sinais vitais, porque ele é técnico de enfermagem, mas não ganha a complementação por técnico, como ele já tem o conhecimento de ser técnico e ele tem empatia pelo paciente, já faz o serviço a mais na residência. Mas se não tem empatia e é um paciente comum para ele, vai trazer a informação para o enfermeiro da equipe ou para o técnico de enfermagem fazer uma visita complementar.* (E10)

Os ACS participantes destacam que a realização desses procedimentos é vista como uma oportunidade para aumentar sua autonomia e valorização profissional. No entanto, também expressam desconforto com o excesso de atribuições burocráticas que lhes são impostas, as quais não resultam no devido reconhecimento de sua profissão. Por outro lado, ambos os grupos de profissionais reconhecem que algumas dessas atribuições pertencem ao técnico de enfermagem e apontam a necessidade de qualificação e/ou capacitação adequada para a realização desses procedimentos.

[...] *Quanto a potencialidades, eu acho o fato deles se sentirem seguros e úteis, a independência de não ficar esperando o enfermeiro* [...] *para realizar essa prática, ter autonomia, a palavra certa é autonomia. Para realizar uma visita com um pouco mais de qualidade. Quando você chega na casa do paciente e leva o aparelho para aferir uma pressão e glicemia ele se sente mais valorizado, como se estivesse em uma consulta* [...]. (E6)

*Eu acho que é de suma importância, se eles dessem cursos* [...] *não simplesmente falar: você vai aferir pressão, fazer um curativo e pronto, não, eles deveriam capacitar os agentes de saúde para que a gente possa exercer, isso é muito importante, eu ficaria lisonjeada de chegar e aferir à pressão do usuário. E muita das vezes ele não poderia vir até aqui passar pelo enfermeiro, pelo médico e já diminuiria a sobrecarga do enfermeiro, do médico, com a visita do agente de saúde.* (A10)

Outro fato importante a ser destacado entre os participantes favoráveis, é o reconhecimento da formação técnica em enfermagem para o exercício legal de tais atribuições. Consideram que, como muitos ACS possuem formação técnica ou são acadêmicos de enfermagem, poderia ocorrer a execução dos procedimentos o que legalizaria as práticas no seu cotidiano. No entanto, abstraem-se de outro problema legal, que seria o desvio de função, pois o profissional permaneceria sendo contratado como agente comunitário de saúde.

*As minhas ACS especialmente, é só uma que não, são técnicas de enfermagem, elas tem curso de técnico e lá tem vários que são técnicos e estão fazendo faculdade de enfermagem e mesmo assim eles não podem fazer, o que eu acho errado, porque se eles vão visitar o paciente não custa nada eles levarem um aparelhinho de pressão, um aparelhinho de glicose, porque se tipo assim, deu uma alteração muito grande na glicose do paciente a gente vai lá faz um insulina, faz um medicação ou vê o prontuário da paciente vê o que ele precisa entendeu? Seria muito bom mesmo, essa ideia é muito boa acha que adiantaria muito o seu trabalho, ia adiantar muito, muito mesmo.* (E8)

[...] *muitos de nós já temos o conhecimento, muitos de nós já somos da área da saúde, muitos já são técnicos de enfermagem, com isso, porém, nem esses têm autorização e não fazem esses tipos de serviços* [...]. (A4)

Os profissionais enfermeiros que são desfavoráveis à inclusão das atribuições apresentadas pela PNAB aos ACS, relataram diversos fatores, dentre eles: questões legais do exercício profissional, desvio de função, a necessidade de formação técnica, fragilidade das condições e cargas de trabalho, preocupações relacionadas a falta de respaldo na supervisão do enfermeiro, lembrando que o ACS não faz parte da equipe de enfermagem, além da descaracterização do papel do ACS na atenção primária.

[...] *inclusive os meus ACS são* [...] *técnicos de enfermagem. Onde eu trabalho, eles recebem uma gratificação da empresa por isso, mas eu falo: “aqui vocês são contratados como ACS* [...] *“não façam porque não é da atribuição de vocês, nós temos a técnica de enfermagem, então ela que vai fazer o curativo, ela que vai administrar a medicação, se eu souber que vocês estão fazendo eu vou dar advertência”* [...]. (E11)

[...] *entendo essas questões trabalhistas, mas elas viriam através de portarias, seriam regulamentadas, eles teriam uma capacitação. Então tem burocracias para isso acontecer, entendeu? E pode acontecer, a questão é o quanto isso vai impactar no trabalho da unidade, na vida pessoal e profissional deles, no nosso controle por conta de situações vividas diariamente na unidade.* (E4)

[...] *O Coren ainda não tem nenhum pronunciamento disso, o COFEN, então antes de ter essa política tem que ter uma discussão entre as entidades da profissão, que no caso é a enfermagem, a responsabilidade de curativo é de quem? Do enfermeiro, então está, o ACS vai fazer e se o ACS usar uma cobertura que lesione ainda mais a ferida do paciente quem é que responde? O enfermeiro que supervisiona o trabalho do ACS? Ok, mas supervisiona o curativo nesse caso? Acho que fica uma lacuna, enquanto fica lacuna eu acho melhor a gente não fazer, porque a gente não tem respaldo* [...]. (E11)

Em relação às questões trabalhistas, os participantes do estudo demonstram preocupação com o exercício ilegal da profissão de técnico de enfermagem e com o desvio de função dos ACS, inclusive para aqueles que possuem formação técnica em enfermagem, pois não foram contratados para exercer essa função.

Os profissionais que se opõem à execução de procedimentos e técnicas pelos ACS argumentam que é necessária a formação técnica em enfermagem para garantir a realização segura e legal dessas atribuições. Assim, apenas capacitação ou qualificação não seriam suficientes diante da complexidade técnica e científica envolvida, sendo fundamental preservar a qualidade do cuidado e a segurança do usuário. Além disso, mesmo que o ACS possua formação técnica e capacitações para realizar esses procedimentos, isso ainda seria considerado um desvio de função. Outro risco apontado está relacionado à supervisão dessas ações, uma vez que falta respaldo ao enfermeiro da equipe, já que o ACS não faz parte da categoria profissional da enfermagem.

*Eu sou contra, porque ele não teve uma formação técnica e não fez um curso técnico em enfermagem, e o máximo que o ACS pode ter é um curso técnico de ACS.* [...] *você tem que ter todo o conhecimento técnico e científico para aquela técnica,* [...] *para ser ACS exige apenas o nível médio, então acho que você colocar uma responsabilidade no profissional de nível médio.* (E11)

[...] *uma pressão mal aferida também você pode prejudicar a vida de alguém, não é só colocar o “esfigmo” e acabou, não, tem toda uma técnica. Então eu sou contra e se puder* [...] *na minha equipe isso não vai acontecer, porque a gente tem um profissional habilitado que o técnico de enfermagem, ele está aí para isso, para fazer os procedimentos que não sejam invasivos, para ele para fazer o curativo, está ali para aferir glicemia, pressão, é isso, eu sou “super” contra.* (E11)

Nesta perspectiva, também foi mencionado pelos enfermeiros desfavoráveis à incorporação das novas atribuições previstas pela PNAB - 2017 para os ACS, a piora das condições e cargas de trabalho já agravadas com a diminuição do número de ACS nas equipes, ocasionando o acúmulo de funções, salários inadequados relacionados às novas atribuições e o impacto negativo na rotina e no processo de trabalho da equipe de Saúde da Família.

[...] *é um profissional que não tem um salário legal, tem um salário de nível médio, você vai dar uma atribuição maior além do que ele já tem e se a gente parar para pensar que não tem aquela obrigatoriedade de números, então vamos supor que um ACS está fazendo um trabalho que seria de dois, três e ainda tem que fazer tudo isso, é adoecedor.* (E1)

[...] *Não e não. Se eu vivo numa bolha eu quero continuar nessa bolha, mas a gente não faz isso lá, de forma nenhuma.* [...] *Acho um acúmulo de funções desnecessário e para supervisionar também seria muito complicado. Pensa bem, uma pessoa vendo um ACS realizando um procedimento, aí essa pessoa mora na comunidade, é um sábado ou um domingo e vão na casa desse ACS para ele realizar esse procedimento, me desculpa, mas as pessoas não têm bom senso, acho que para a vida pessoal deles também seria bem ruim. Já é ruim e acho que pioraria exponencialmente.* (E4)

O participante E11 argumenta que a PNAB - 2017coloca os ACSs como executores de tarefas, contribuindo para a mecanização das suas ações, reforçando o modelo biomédico e a descaracterização do seu papel na APS.

[...] *então não é só o ato de você chegar e mecanizar tudo o que eu acho é quem formulou isso pensa dessa forma, que é só uma pressão, até para você aferir a pressão tem que saber, você tem que saber orientar o paciente, “o senhor veio andando? Tem que esperar, se o senhor fumou, você bebeu água?* [...]". *então eu acho que foi uma forma muito assim de pensar no ACS como executor, só executou sem pensamento crítico* [...]. (E11)

Além disso, encontramos relatos dos participantes do estudo, referindo as resistências dos ACS à incorporação das novas atribuições às suas práticas na equipe de Saúde da Família. Consequentemente, identificou-se que parte dos participantes do estudo que rejeitam a PNAB - 2017 são adeptos à política anterior; e reforçam que aferir pressão arterial, glicemia, temperatura e realizar curativos são procedimentos da enfermagem e que não pertencem à função do ACS, apontando novamente a sobrecarga de trabalho no acompanhamento dos usuários.

[...], *mas eles são totalmente contra, 99% são contra, já foi falado em reunião todo mundo deu contra, foi uma confusão terrível, ninguém aceita isso de ir para a casa do paciente e aferir.* (A3)

*Essa atribuição é do técnico de enfermagem não do agente de saúde, é uma atribuição dele, porque o técnico tem um dia na semana que o dia de visita dele na comunidade, no território* [...] *eu acho que isso não é atribuição do ACS* [...]. (A6)

Os enfermeiros entrevistados ressaltam que os ACS são importantes para o território, no estabelecimento dos vínculos, na busca ativa, no acolhimento, na detecção de demandas de saúde dos usuários, ou seja, na sua real função. Assim, essas novas atribuições distanciam os ACS da sua origem, o que pode gerar lacunas no cuidado e repercussões negativas no processo de trabalho das equipes.

[...] *preciso da percepção, não que ele faça um curativo, eu acho que a gente peca um pouco em relação às atribuições porque não é o que tá me fazendo falta, não faz falta ele fazer isso, faz falta ele realmente fazer um acolhimento de excelência, que para mim ele nasceu para isso, para poder saber identificar determinado paciente de forma individual, não é porque um paciente assim é assim e outro que chegou com a mesma história que eles vão ser tratados iguais, porque a gente tem que entender que cada ser humano ele vai ser único.* [...] *vocês precisam se fazer importantes, porque para mim são e são muito, mas se não tivesse ACS na minha equipe eu não ia saber trabalhar porque são eles que filtram as demandas que chegam, eles que filtram as mentiras que chegam, eles conseguem identificar urgências.* (E9)

## DISCUSSÃO

Em setembro de 2017, o Conselho Federal de Enfermagem (COFEn) solicitou esclarecimentos ao Ministério da Saúde (MS) acerca da realização de procedimentos técnicos de enfermagem pelos agentes comunitários de saúde, preconizados pela PNAB-2017^(14)^. No mesmo período, ocorreu uma plenária do COFEn que recebeu representantes do Departamento de Atenção Básica do MS e do Conselho Nacional de Secretários de Saúde (CONASS) para discutir as novas atribuições dos agentes comunitários de saúde, atividades asseguradas pela Lei 7.498/1986^(15)^, que regulamenta o exercício profissional da enfermagem. Contudo, o então Ministro da Saúde, Ricardo Barros, afirmou que somente agentes com formação técnica em enfermagem realizariam tais atribuições. Essa declaração gerou críticas dos conselheiros, uma vez que não foi garantida a exigência da habilitação técnica em enfermagem^(14)^.

Em 2018, o COFEn recebeu a Confederação Nacional dos Agentes Comunitários de Saúde e Agentes de Combate às Endemias (CONACS) para debater o desvio de função imposto pelo MS através da PNAB-2017, afirmando que essa política descaracterizava as atribuições dos ACS e visava usá-los como técnicos de enfermagem, ou até mesmo extinguir a profissão, enfraquecendo sua atuação no SUS e na APS, o que poderia representar riscos à saúde da população^(16)^.

É importante destacar outras duas portarias do MS que, devido à pressão e resistência dos profissionais, foram posteriormente revogadas: a Portaria nº 958/2016^(17)^, que previa a substituição dos ACS por auxiliares ou técnicos de enfermagem, e a Portaria nº 83/2018^(18)^, que propunha a formação técnica em enfermagem para agentes comunitários de saúde e agentes de combate a endemias, por meio do Programa de Formação Técnica em Enfermagem para Agentes de Saúde (PROFAGS).

Segundo a Associação Brasileira de Enfermagem (ABEn), o Programa PROFAGS descaracteriza as funções profissionais na APS, ao flexibilizar e desfigurar os processos de trabalho na Atenção Básica, além de ameaçar os postos de trabalho dos agentes comunitários de saúde^(19)^. O grupo de trabalho dos Agentes de Saúde do COFEn emitiu um parecer contrário ao PROFAGS, afirmando que, caso os ACS fossem capacitados em curso técnico de enfermagem, deveriam ser contratados para essa função e não como agentes comunitários de saúde^(20,21)^.

Apesar dessas portarias terem sido revogadas devido à resistência de profissionais e entidades científicas como a ABEn, CONACS e o COFEn, as atribuições foram consolidadas na Portaria GM/MS nº 2.436/2017^(4)^. Após análise dos papéis e legislações referentes aos ACS e técnicos de enfermagem, constatou-se que as portarias evidenciam a ideia de que os ACS não seriam necessários na APS, por serem considerados pouco resolutivos no combate às doenças crônicas-degenerativas. Sugere-se que o técnico de enfermagem atenderia melhor essas demandas, tornando os ACS dispensáveis na composição mínima das equipes^(22)^. Essa constatação é reforçada pela PNAB-2017, que torna facultativa a presença dos ACS nas equipes de Atenção Básica^(4)^.

Concordamos que as duas portarias reforçam as ideias da PNAB-2017, pois foram implementadas evidenciando um audacioso planejamento de sucateamento do SUS, sempre direcionadas para a tensão entre o público e o privado, tratando a saúde como mercadoria e a Atenção Básica de forma seletiva e reduzida, voltada para a lógica do modelo biomédico e hegemônico^(23)^.

A execução de atribuições específicas da enfermagem pelos ACS criará problemas administrativos, jurídicos e de gestão para a APS, como a insuficiência de ACS ou técnicos de enfermagem nas unidades, profissionais essenciais para o funcionamento das atividades fundamentais. Essas são algumas das barreiras que acabam gerando desvios de função e aprisionamento do ACS na unidade de saúde^(19)^.

De acordo com a Lei do Exercício Profissional da Enfermagem (Lei 7.498/1986)^(14)^, o enfermeiro supervisiona e é eticamente responsável pelas ações dos auxiliares de enfermagem, técnicos de enfermagem e parteiras, mas o ACS não é parte da equipe de enfermagem. No entanto, no que se refere às atribuições dos ACS e dos agentes de combate às endemias (ACE), a PNAB - 2017 define atribuições comuns e específicas para ambas as categorias, incluindo a supervisão por profissionais de nível superior, como o enfermeiro da equipe^(4)^.

O parecer técnico do COREn-DF Nº 051/CTA/2022, em resposta a questionamentos sobre quem deve supervisionar as atividades dos agentes de saúde pública na APS e sobre o ensino e realização de técnicas e procedimentos de enfermagem, reafirma que a supervisão deve ser realizada em conjunto com a equipe multiprofissional. No entanto, de forma ambígua, o parecer conclui permitindo que os ACS realizem técnicas e procedimentos de acordo com as diretrizes da PNAB-2017, o que contraria o posicionamento do COFEn e da ABEn^(20)^.

Diante dos diversos ataques à PNAB, especialmente no que se refere à presença facultativa dos ACS nas equipes de Atenção Básica, à redução de sua contingência na ESF, e à inclusão de novas atribuições para esses trabalhadores, que já estão sobrecarregados com práticas de educação e promoção da saúde, corre-se o risco de retrocessos no processo de trabalho da APS. Isso pode ocultar as realidades de vida que influenciam os processos de saúde-doença^(24,25)^.

Essa perspectiva também é mencionada em outro estudo, que defende a inclusão de técnicos e auxiliares de enfermagem nas equipes de Saúde da Família. Ressalta-se, ainda, que ao agregar as práticas da enfermagem ao trabalho dos ACS, ocorre a ampliação de procedimentos técnicos no território, em detrimento das atividades essenciais realizadas pelos ACS^(7)^.

O ACS, como integrante da equipe interprofissional, contribui de formas distintas nas ações de promoção, prevenção, investigação, diagnóstico e recuperação da saúde da população. Esse papel reforça a necessidade de uma equipe de saúde coesa, com iniciativas intersetoriais para atender às diversas demandas do território em que atua^(24)^.

Ao longo dos anos, as práticas dos ACS foram aprimoradas, o que gerou novos desafios, incluindo o enfrentamento de resistências e a luta por melhores condições de trabalho, além da manutenção de seu papel original voltado para a promoção e prevenção no território. Segundo os próprios ACS, essas ações eram vistas como responsáveis por proporcionar reconhecimento e respeito dos usuários. No entanto, suas práticas de saúde estão cada vez mais distantes dos usuários, devido ao excesso de serviços administrativos e à permanência prolongada no interior das unidades. Esse afastamento do território impede o acompanhamento adequado das famílias, o que descaracteriza o trabalho dos ACS e compromete a proposta da ESF^(26)^.

É notório que os interesses privatistas e mercantilistas, que se tornaram hegemônicos no governo federal entre 2015 e 2022, influenciaram a formulação de um conjunto de projetos, emendas constitucionais e manifestações públicas, com o objetivo de enfraquecer o SUS e substituí-lo por serviços privados de baixa qualidade, baseados em “outros” modelos de assistência, com “cestas” de serviços limitados, como ocorre com os seguros de saúde, que seguem o modelo biomédico^(19)^.

A transferência de funções do técnico de enfermagem para o ACS também aponta para a redução de custos, uma vez que a manutenção desses trabalhadores é menos onerosa do que aumentar o contingente de técnicos, favorecendo a lógica capitalista do setor de saúde e precarizando os serviços e as relações de trabalho.

Sob a hegemonia da racionalidade neoliberal, a lógica privatista tem forçado sua ampliação dentro do SUS, progressivamente integrando-se à estrutura do sistema. Ideias ligadas às relações de mercado têm sido incorporadas aos desenhos das políticas e aos processos de gestão, impondo limites à universalização do direito, à concepção ampliada de saúde e à base de financiamento da seguridade social^(25)^.

A relação entre poder e saber, ligada à prática social dos ACS, tem se transformado com a institucionalização desses trabalhadores, afastando-os de suas funções, papéis e perfis originais, sobretudo pelas influências tecnicistas na saúde, onde o saber é modificado e o poder se torna objeto de disputa. Assim, a inserção pública, a criação da profissão de ACS e a regulamentação de seu trabalho reconfiguraram suas funções, papéis e atribuições^(27)^.

A formação do ACS está centrada no saber popular, com o objetivo de promover a saúde a partir da participação popular, o que potencializa a compreensão de que a saúde é um direito social constituído pelos determinantes sociais da saúde. Dessa forma, sua formação não possui uma base histórica voltada para o modelo biomédico tradicional^(28)^.

Nesse contexto, a precarização da atenção à saúde se intensifica com a substituição desses profissionais por auxiliares ou técnicos de enfermagem, conforme previsto na Portaria 958/2016^(17)^, e com o surgimento da PNAB-2017, que torna a presença do ACS facultativa nas equipes de Atenção Básica, reduzindo sua participação nas equipes de Saúde da Família e incorporando procedimentos de enfermagem como atribuições dos ACS.

As repercussões da PNAB-2017, com as contradições e inquietações identificadas neste estudo, revelam a realidade concreta das práticas de saúde dos ACS. Entende-se que a adoção do modelo neoliberal nos serviços de saúde está ligada à precarização dos trabalhadores da saúde, incluindo ACS e enfermeiros, com a adoção de baixos salários, condições precárias de trabalho, precarização das relações trabalhistas, e diminuição das equipes assistenciais^(29)^, o que contribui para a descaracterização das práticas de saúde desses profissionais.

Trata-se de um movimento político que esvazia a saúde a partir da desvalorização de seus trabalhadores, colocando em risco a proposta de vínculo terapêutico contínuo e a manutenção dos princípios de territorialidade e cuidado comunitário, que são pilares da APS e que se perdem com a reorganização do sistema^(29)^.

Essas mudanças refletem as contradições nas relações dos ACS com o trabalho, polarizando e confundindo as opiniões dos próprios profissionais sobre suas origens, papéis e atribuições, além de ameaçar sua existência e importância nos serviços de saúde. Pode-se associar esse esvaziamento da profissão à concretização do neotaylorismo, que exige maior escolaridade/qualificação para algumas profissões, como no caso das atribuições técnicas de enfermagem conferidas aos ACS. No entanto, percebe-se que, enquanto algumas profissões estão adquirindo novas atribuições, outras estão sendo esvaziadas^(30)^.

### Limitações do estudo

Aponta-se como limitação do estudo a não inclusão das outras Áreas Programáticas do município do Rio de Janeiro, além do pequeno número de participantes da pesquisa na AP selecionada, agravado pelo contexto de crise sanitária causada pela pandemia de COVID-19. Outra limitação foi a não inclusão de outros profissionais, gestores e usuários da ESF neste estudo. No entanto, apesar dessas limitações, este estudo oferece um panorama da percepção dos profissionais em relação às novas atribuições do ACS. Considera-se que há riscos à saúde da população quando se permite que profissionais realizem procedimentos técnicos sem a devida formação e competência, fragilizando a APS e a ESF.

### Contribuições para a área

Este estudo revela a necessidade de refletir e aprofundar as pesquisas, utilizando outros métodos, sobre as atribuições e contribuições do ACS nas equipes de Saúde da Família, reforçando e resgatando o papel deste profissional nesse campo de atenção. É importante que os profissionais de enfermagem atuem na fiscalização da Lei do Exercício Profissional da Enfermagem e promovam discussões, reflexões e mobilizações para que a PNAB seja reestruturada conforme os princípios do SUS. O objetivo deve ser o fortalecimento e a expansão da APS, priorizando a ESF e, principalmente, respeitando o escopo de atribuições, competências e habilidades de acordo com a formação dos profissionais da equipe de saúde.

## CONSIDERAÇÕES FINAIS

Diante da polarização entre enfermeiros/as e ACS favoráveis e contrários à inclusão de atribuições do técnico de enfermagem para o ACS, é fundamental ampliar o debate sobre o reconhecimento social e econômico do trabalho, que justifica a concordância com as novas atribuições por parte de alguns.

É necessário considerar os diferentes contextos e as contradições presentes nas práticas de saúde dos ACS. A necessidade de cumprir tarefas predeterminadas acaba por moldar novas formas de oferta de serviços, que nem sempre consideram as perdas de direitos sociais, como o direito à saúde. Parece-nos que não houve uma reflexão aprofundada sobre a qualidade do cuidado, a segurança do paciente, o acesso e a integralidade da atenção. Comentários como “não ficar sobrecarregado” e “dividir direitinho os usuários entre os ACS” são respostas a uma política que leva os profissionais de saúde a aceitar o que é imposto, demonstrando nossa baixa participação nos processos decisórios em saúde e nossa “pouca força” na defesa da saúde como um direito de todos.

A política vigente posiciona os ACS como meros executores de tarefas, contribuindo para a mecanização das ações, precarizando as condições de trabalho dos ACS, reforçando o modelo biomédico e descaracterizando seu papel na promoção da saúde na APS.
